# Depressive symptoms and their associations with positive psychosocial factors among medical students

**DOI:** 10.1097/MD.0000000000047333

**Published:** 2026-01-23

**Authors:** Eun Hyun Seo, Seung-Gon Kim, Seon-Cheol Park, Hyung-Jun Yoon

**Affiliations:** a Premedical Science, Chosun University College of Medicine, Gwangju, Republic of Korea; b Department of Psychiatry, Chosun University College of Medicine, Gwangju, Republic of Korea; c Department of Psychiatry, Hanyang University College of Medicine, Seoul, Republic of Korea.

**Keywords:** depression, ego-resiliency, medical students, self-esteem, social support

## Abstract

The role of positive psychosocial factors in depression among medical students has been insufficiently studied. This study investigated the prevalence of depression and its association with positive psychosocial factors among Korean medical students. A total of 408 medical students completed a self-report questionnaire on sociodemographic characteristics, self-esteem, ego-resiliency, social support, and depression. Depressive symptoms were assessed using the Beck Depression Inventory (BDI), with scores of 16 or higher indicating the presence of depression. Fifty-five (13.5%) participants had depression. Medical students with depression showed significantly lower self-esteem, ego-resiliency, and social support than those without depression. Hierarchical regression analyses indicated that when sociodemographic factors were controlled for, self-esteem, ego-resiliency, and social support were strongly associated with lower BDI scores. Female sex and preclinical years were associated with higher BDI scores. While the findings suggest that positive psychosocial factors could play a crucial protective role against depression in medical students, the cross-sectional design limits causal inference. Longitudinal studies are required to confirm these associations.

## 1. Introduction

Medical students undergo intense education processes to become competent medical professionals capable of providing better medical care and promoting public health. Compared with other majors, the training time for medical students to acquire the necessary knowledge and skills is longer, and the physical and psychological burden during medical education is significantly greater. A substantial proportion of medical students experience psychological distress, such as depression and burnout, due to a variety of factors including excessive academic workloads, fear of failing, extreme competition, and sleep deprivation.^[1,2]^ To cope with stress and depression, medical students may engage in harmful and potentially addictive behaviors such as hazardous alcohol consumption and excessive Internet or smartphone use.^[3]^ A previous study showed that depression and anxiety were more prevalent in medical students than in the general population and age-matched peers.^[2]^

Depression is a common mental disorder and an important public health problem because of its high prevalence and negative impact on psychosocial functioning.^[4]^ According to the Global Burden of Disease study, depression constituted the largest proportion of disability-adjusted life years caused by mental disorders in 2019.^[5]^ Depression has been emphasized as an important mental health issue among medical students because depressive symptoms have a negative impact on their quality of life and may lead to suicide in severe cases.^[6]^ Furthermore, medical students with depression show significantly poorer academic performance than those without depression.^[7]^ As the mental health of medical students is closely related to the future quality of healthcare,^[8]^ it is important to implement intervention programs for the prevention and treatment of mental disorders during medical education. Despite serious suffering due to depression, medical students are less likely to utilize psychiatric care than the general population.^[9]^ Stigmatization or fear of confidentiality issues may act as obstacles to seeking professional mental health services for medical students.

Sex and several factors related to medical education, such as workload and lack of feedback, are significantly associated with depression among medical students.^[10]^ In a longitudinal study of medical students, personal factors, including anxiety and relationship problems, were found to be relevant to the persistence of depression.^[11]^ In the past decade, psychosocial factors derived from positive psychology, which primarily focuses on the positive aspects of life, have received notable attention as an alternative approach to reducing depression. These factors include self-esteem, ego-resiliency, and social support and have been investigated as protective factors against depression. Self-esteem is defined as an overall indicator of self-evaluation that involves the cognitive appraisal of self-worth and affective experiences of the self.^[12]^ Ego-resiliency refers to flexible and effective coping with and adaptation to adverse and ever-changing situations.^[13]^ Social support refers to the material resources and emotions available to a person through interpersonal relationships. High self-esteem and ego-resiliency have been shown to have protective effects against depression among adolescents.^[14,15]^ It was also found that close relationships and perceived support were negatively related to depression in a community sample, suggesting that social support may be an important factor in reducing depressive symptoms.^[16]^ Thus, it is possible that these positive psychosocial factors may show a beneficial effect on depressive symptoms in medical students.

Previous studies on depression in medical students have primarily examined its prevalence and associated sociodemographic and academic factors. Despite meaningful relationships between positive psychosocial factors and depressive symptoms, few studies have examined the association between these factors and depression among medical students. Cultural values such as hierarchical educational structure, academic perfectionism, and reluctance to express emotional vulnerability may exacerbate stress and discourage help-seeking behaviors among Korean medical students.^[17]^ These pressures could intensify depression risk and warrant targeted investigation. Furthermore, there are limited studies on the prevalence and factors related to depression in this population in Korea. This study aimed to investigate the impact of self-esteem, ego-resiliency, and social support on depressive symptoms among medical students after adjusting for sociodemographic variables related to depression.

## 2. Materials and methods

### 2.1. Participants

This study was conducted among the medical students at Chosun University. The curriculum of Chosun University is 6 years, with the first 2 years comprising the premedical curriculum. The 4-year medical curriculum is composed of 2 years of preclinical curriculum followed by 2 years of clinical curriculum. Major and minor clinical rotations are arranged during the 3rd and 4th years of education, respectively. Convenience sampling was used to gather participants. The data collection procedures have been described previously.^[18]^ Participants completed a self-report questionnaire comprising items on sociodemographic characteristics, depressive symptoms, self-esteem, ego-resiliency, and social support. No monetary compensation was provided to participants.

### 2.2. Measures

#### 2.2.1. Depressive symptoms

The severity of depressive symptoms was evaluated using the Beck Depression Inventory (BDI), which is composed of 21 items.^[19]^ Each item is rated on a 4-point (0–3) scale, with higher scores indicating more severe depression. The Korean version of the BDI has been standardized and a cutoff score of 16 for depression has been suggested.^[20]^ Participants with a score of 16 or higher were considered to have depression.

#### 2.2.2. Self-esteem

Self-esteem was evaluated using the Rosenberg Self-Esteem Scale (RSES), which consists of 10 items.^[21]^ Each item of the RSES is rated on a 4-point (1–4) scale with higher scores indicating higher self-esteem. The reliability and validity of the Korean version of the RSES have been previously confirmed.^[22]^

#### 2.2.3. Ego-resiliency

Ego-resiliency was measured using the Ego-Resiliency Scale (ERS).^[13]^ The ERS consists of 14 items assessing flexibility, curiosity, generosity, and social skills. Each item is rated on a 4-point (1–4) scale, with a higher score reflecting a higher level of ego-resiliency. The Korean version of the ERS has been standardized.^[23]^

#### 2.2.4. Social support

The Duke University of North Carolina Functional Social Support Questionnaire (Duke-UNC FSSQ) was used to evaluate degree of social support. Eight items of the Duke-UNC FSSQ, consisting of 2 subscales (confidant and affective support), were used to calculate the mean social support score.^[24]^ Each item is rated on a 5-point (1–5) scale with a higher score reflecting a higher level of overall social support. The reliability and validity of the Korean version of the Duke-UNC FSSQ have been confirmed.^[25]^

### 2.3. Ethics statement

This study conformed to the provisions of the Declaration of Helsinki and was approved by the Institutional Review Board of the Chosun University (IRB No. 2-1041055-AB-N-01-2019-23). Written consent was obtained after the purpose and procedures of the study were explained by a researcher with no relationship with the medical students. Participants were made aware of the voluntary nature of the survey and that their information was anonymous and confidential.

### 2.4. Statistical analysis

Participants were grouped either into a depression or non-depression group based on their BDI scores, with a cutoff score of 16. Continuous variables were checked for normal distribution using the Kolmogorov–Smirnov test. Because all continuous data were non-normally distributed, nonparametric tests were performed. Group comparisons of sociodemographic characteristics and mean RSES, ERS, and Duke-UNC FSSQ scores were performed using the Mann–Whitney *U* test for continuous variables and the Chi-square test or Fisher exact test for categorical variables. The Spearman rank correlation test was performed to examine the relationship between depression severity and psychosocial characteristics. To assess the adjusted associations with depressive symptoms, hierarchical regression analyses were conducted (method entry) for the BDI. Factors significantly associated with depression were entered into the regression models as independent variables. Sex, age, and school year were included as predictors. Self-esteem, ego-resiliency, and social support were entered in the second model. Subsequently, sex and school year were used as subgroup variables to test for differences in RSES, ERS, and Duke-UNC FSSQ scores between the depression and non-depression groups. All tests were two-sided, with the significance level set at *P* < .05. All analyses were performed using IBM SPSS for Windows (version 27.0; IBM Corp., Armonk).

## 3. Results

### 3.1. Prevalence of depression and related sociodemographic characteristics

**Figure 1.**
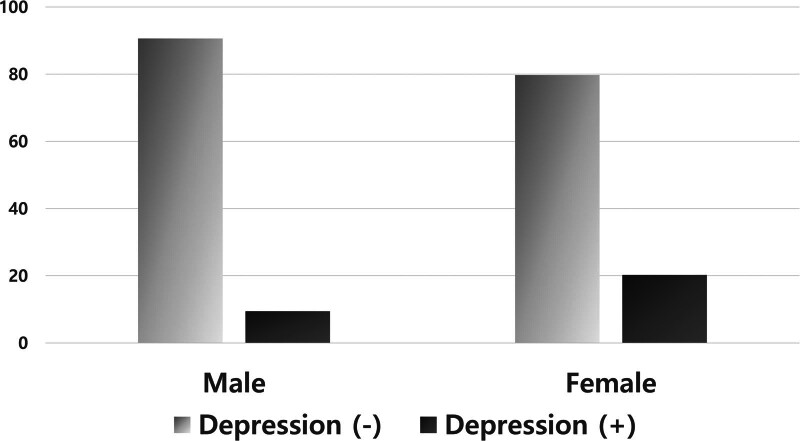
Comparison of depressive symptoms according to sex. Female students (20.3%) showed a significantly higher rate of depression (Beck Depression Inventory ≥ 16) than male students (9.4%).

A total of 418 medical students participated in this study. Excluding 10 invalid questionnaires (those with >25% unanswered questions), 408 completed self-report questionnaires were analyzed. Their ages ranged from 20 to 45 years, and the mean age was 26.3 ± 4.4 years. Of the 408 students, 255 (62.5%) were male and 153 (37.5%) were female. The prevalence of depression was 13.5% (N = 55). The participants with depression were significantly younger than those without depression. Female students (n = 31, 20.3%) showed a significantly higher rate of depression than male students (n = 24, 9.4%), as shown in Figure 1. Depression was significantly higher among students undergoing preclinical education (1st and 2nd years) than among those undergoing clinical education (3rd and 4th years). However, there were no significant differences in the other sociodemographic variables between the 2 groups. The sociodemographic characteristics of the participants and comparisons by depression status are shown in Table 1.

**Table 1:** Group comparisons of sociodemographic factors according to the presence of depression.

Variables	Depression			*r* or *χ*^2^	*P*
	No 353 (86.5)	Yes 55 (13.5)	Total 408 (100.0)		
Age (yr)	26.5 ± 4.4	25.2 ± 3.9	26.3 ± 4.4	‐.10	.044*
Sex					
Male	231 (65.4)	24 (43.6)	255 (62.5)	9.65	.002
Female	122 (34.6)	31 (56.4)	153 (37.5)		
School year					
First-year	93 (26.3)	27 (49.1)	120 (29.4)		
Second-year	77 (21.8)	17 (30.9)	94 (23.0)	20.25	<.001
Third-year	94 (26.6)	6 (10.9)	100 (24.5)		
Fourth-year	89 (25.2)	5 (9.1)	94 (23.0)		
Marital status					
Never married	333 (94.3)	55 (100.0)	388 (95.1)		.090†
Married	20 (5.7)	0 (0.0)	20 (4.9)		
Residence					
With family	112 (31.8)	14 (25.5)	126 (31.0)		
Dormitory	13 (3.7)	3 (5.5)	16 (3.9)	1.15	.562
Alone	227 (64.5)	38 (69.1)	265 (65.1)		
Subjective SES					
High	68 (19.3)	12 (21.8)	80 (19.6)		
Middle	258 (73.1)	36 (65.5)	294 (72.1)	2.02	.365
Low	27 (7.6)	7 (12.7)	34 (8.3)		

Data are presented as mean ± standard deviation or N (%).

SES = socioeconomic status.

* Statistical significance was tested using the Mann–Whitney *U* test.

† Statistical significance was tested using Fisher exact test.

### 3.2. Positive psychosocial factors according to the presence of depression

The total RSES and ERS scores were significantly lower in the depression group than in the non-depression group. Similarly, the Duke-UNC FSSQ total scores were significantly lower in the depression group than in the control group. Furthermore, both Duke-UNC FSSQ subscale scores were significantly lower in the depression group than in the non-depression group. The group comparisons of positive psychosocial factors are shown in Table 2.

**Table 2:** Group comparisons of self-esteem, ego-resiliency, and social support based on depression.

Variables	Depression			*r*	*P* *
	No	Yes	Total		
Self-esteem					<.001
RSES	32.4 ± 4.3	23.35 ± 6.7	31.2 ± 5.6	‐.44	
Ego-resiliency					<.001
ERS	39.4 ± 5.5	33.6 ± 6.6	38.6 ± 6.0	‐.29	
Social support					
Duke-UNC FSSQ total	34.2 ± 5.7	26.1 ± 8.3	33.1 ± 6.7	‐.34	<.001
Duke-UNC FSSQ component					
Confidant support	20.8 ± 4.0	15.6 ± 5.5	20.1 ± 4.6	‐.32	<.001
Affective support	13.4 ± 2.2	10.5 ± 3.3	13.0 ± 2.6	‐.33	<.001

Data are presented as mean ± standard deviation or N (%).

Duke-UNC FSSQ = Duke University of North Carolina Functional Social Support Questionnaire, ERS = Ego-Resiliency Scale, RSES = Rosenberg Self-Esteem Scale.

* Statistical significance was determined using the Mann–Whitney *U* test.

### 3.3. Correlations between depressive symptoms and positive psychosocial factors

The total BDI scores exhibited a significant negative correlation with the RSES, ERS, and Duke-UNC FFSQ total scores. Spearman ρ for depressive symptoms with self-esteem, ego-resiliency, and social support are shown in Table 3.

**Table 3:** Spearman rank correlations between depressive symptoms and self-esteem, ego-resiliency, and social support.

Variables	1	2	3	4
1. BDI	1			
2. RSES	‐.592*	1		
3. ERS	‐.379*	.435*	1	
4. Duke-UNC FSSQ	‐.480*	.430*	.240*	1

BDI = Beck Depression Inventory, Duke-UNC FSSQ = Duke University of North Carolina Functional Social Support Questionnaire, ERS = Ego-Resiliency Scale, RSES = Rosenberg Self-Esteem Scale.

* *P* < .001.

### 3.4. Association between positive psychosocial factors and depressive symptoms

The results of the hierarchical regression analysis are summarized in Table 4. In the first model, female sex, 1st year, and 2nd year were positively associated with the BDI total scores. Together, these sociodemographic factors accounted for 9.3% of the variance in the total BDI scores. After controlling for sociodemographic factors, self-esteem, ego-resiliency, and social support were negatively associated with the total BDI scores in the second model. This model explained an additional 44.5% of the variance in the total BDI scores. Although the associations between female sex and 1st- and 2nd-year BDI total scores were still significant, the impacts of these factors were lower in the final model than in the previous model.

**Table 4:** Hierarchical regression analysis to predict depressive symptom severity.

Variables	Model 1			Model 2		
	Stand β*	*t*	*P*	Stand β*	*t*	*P*
Sex (ref: male)						
Female	.157	3.288	.001	.105	3.002	.003
Age	.069	1.206	.229	.027	.661	.509
School year (ref: 4th-year)						
First-year	.283	3.930	<.001	.148	2.831	.005
Second-year	.285	4.392	<.001	.139	2.946	.003
Third-year	.017	.272	.786	‐.028	‐.633	.527
Self-esteem						
RSES scores				‐.475	‐11.071	<.001
Ego-resiliency						
ERS scores				‐.091	‐2.322	.021
Social support						
Duke-UNC FSSQ scores				‐.243	‐6.065	<.001
Adjusted *R*^2^	.093			.538		
Adjusted *R*^2^ change				.445		
	*F* = 11.436, *P* < .001			*F* = 68.616, *P* < .001		

Duke-UNC FSSQ = Duke University of North Carolina Functional Social Support Questionnaire, ERS = Ego-Resiliency Scale, Ref = reference group, RSES = Rosenberg Self-Esteem Scale.

* Standardized beta.

### 3.5. Subgroup analysis using sex stratification

Subgroup analysis using sex stratification indicated that self-esteem, ego-resiliency, and social support were significantly associated with non-depressive status in both male and female students (Table 5).

**Table 5:** Subgroup analysis using sex stratification.

Variables	Male				Female			
	Depression		*r*	*P* *	Depression		*r*	*P* *
	No	Yes			No	Yes		
RSES scores	32.6 ± 4.2	22.5 ± 6.7	‐.32	<.001	32.0 ± 4.5	24.0 ± 6.8	‐.29	<.001
ERS scores	40.0 ± 5.4	33.0 ± 7.3	‐.22	<.001	38.3 ± 5.6	33.9 ± 6.2	‐.16	.001
Duke-UNC FSSQ scores	33.6 ± 5.9	22.7 ± 7.6	‐.29	<.001	35.2 ± 5.4	28.7 ± 8.0	‐.22	<.001

Duke-UNC FSSQ = Duke University of North Carolina Functional Social Support Questionnaire, ERS = Ego-Resiliency Scale, RSES = Rosenberg Self-Esteem Scale.

* Statistical significance was determined using the Mann–Whitney *U* test.

### 3.6. Subgroup analysis using school year stratification

The subgroup analysis using school year stratification indicated that self-esteem, ego-resiliency, and social support were significantly associated with non-depression status among all school-year students, except for the association between ego-resiliency and depression status among 3rd-year students (Table 6).

**Table 6:** Subgroup analysis using school year stratification.

Variables	First-year				Second-year			
	Depression		*r*	*P* *	Depression		*r*	*P* *
	No	Yes			No	Yes		
RSES scores	32.6 ± 4.5	23.2 ± 6.7	‐.29	<.001	31.8 ± 4.7	24.3 ± 7.2	‐.20	<.001
ERS scores	40.4 ± 5.8	33.7 ± 6.1	‐.23	<.001	38.7 ± 5.5	34.2 ± 6.9	‐.12	.016
Duke-UNC FSSQ scores	33.7 ± 6.1	28.4 ± 8.4	‐.15	.002	32.8 ± 6.1	23.9 ± 6.9	‐.21	<.001

Variables	Third-year				Fourth-year			
	Depression		*r*	*P* *	Depression		*r*	*P* *
	No	Yes			No	Yes		
RSES scores	32.4 ± 4.1	22.3 ± 8.1	‐.15	.003	32.7 ± 4.0	22.0 ± 4.6	‐.18	<.001
ERS scores	38.9 ± 5.4	33.8 ± 8.3	‐.09	.072	39.5 ± 5.3	30.2 ± 7.4	‐.13	.008
Duke-UNC FSSQ scores	34.8 ± 5.8	20.5 ± 9.9	‐.16	.001	35.2 ± 4.7	27.8 ± 7.5	‐.12	.014

Duke-UNC FSSQ = Duke University of North Carolina Functional Social Support Questionnaire, ERS = Ego-Resiliency Scale, RSES = Rosenberg Self-Esteem Scale.

* Statistical significance was determined using the Mann–Whitney *U* test.

## 4. Discussion

This study examined the relationship between positive psychosocial factors and depressive symptoms among medical students, after adjusting for sociodemographic covariates. We found that self-esteem, ego-resiliency, and social support were negatively associated with depression severity. However, female sex and the 1st and 2nd years were positively associated with the severity of depression.

In the present study, self-esteem and ego-resiliency were independently associated with lower levels of depression, even after controlling for sociodemographic factors, which is consistent with previous studies.^[26,27]^ Furthermore, self-esteem and ego-resiliency were related to non-depression, irrespective of sex and school year, except for the relationship between ego-resiliency and depression in 3rd-year students. Particularly, self-esteem was found to be the strongest predictor of lower depression severity. In longitudinal studies, adolescents with high self-esteem or ego resiliency were found to be less likely to experience depression, suggesting that both self-esteem and ego resiliency may have long-term protective effects against depression.^[14,15]^ People with lower self-esteem tend to ruminate about unfavorable attributes or deficits rather than strengths. On the contrary, high self-esteem and resilience enable people to manage stressful situations well and also cope effectively with challenges when negative life events are encountered. Previous studies have shown that people with high self-esteem and resilience tend to find positive meaning when faced with problems and share psychological resources.^[28]^ Self-esteem and ego-resiliency are related to positive emotions that prevent mental health problems by controlling the negative effects of stress.^[28,29]^ These results are consistent with previous findings on students’ mental health, extending them to a Korean medical student population. For example, Hamaideh et al^[30]^ reported that perceived stress among nursing students was significantly influenced by psychosocial factors such as satisfaction. Consistent with our results, students with higher satisfaction exhibited lower stress levels, suggesting that positive personal traits can buffer psychological distress across healthcare training settings. Although their study focused on nursing students in Jordan, the shared stress-inducing nature of academic environments suggest these protective factors are relevant across cultures and healthcare disciplines.

The quality of social interaction is an important resource for psychological well-being across all stages of adulthood.^[31]^ In this study, social support was found to have a significant negative association with depression. Similar to self-esteem and ego-resiliency, social support was significantly associated with non-depressive status regardless of sex or school year. Moreover, medical students with depression experienced lower levels of confidant and affective components of social support than those without depression. These findings are consistent with those of previous studies.^[32,33]^ Emotional care and family support are important resources for medical students to overcome stressful processes during medical education. Medical students have reported that meaningful relationships with family members, friends, and teachers improve their quality of life.^[34]^ Our results support the viewpoint that social support is a valuable resource, and suggest that it plays a key protective role against depression among medical students. Hamaideh et al^[30]^ noted the importance of interpersonal and family relationships in mitigating stress among nursing students. Hierarchical regression analyses confirmed that the negative effects of female sex and school year (1st and 2nd-year) on depression were reduced in the presence of self-esteem, ego-resiliency, and social support. This result indicates that positive psychosocial factors may act as a buffer against negative sociodemographic factors among medical students.

The prevalence of depression was 13.5% in our sample; a finding that is similar to previous studies with Swedish and Malaysian medical students.^[10,35]^ A nationwide study by Roh et al^[36]^ surveyed >7300 medical students across 36 of Korea’s 41 medical schools, reporting a depression prevalence of 9.4% (BDI ≥ 16) and low rates of clinical diagnosis or treatment (8.9% and 9.7%, respectively) among those screening positive. In comparison, our single institution sample showed a slightly higher prevalence (13.5%), likely owing to site-specific characteristics and the use of convenience sampling. However, the rate was lower than that reported in medical students from Ireland and the United States^[27,37]^ and 27.2% in a previous meta-analysis.^[38]^ Koreans with depression are more likely to express somatic symptoms but less likely to express depressed moods. Therefore, cross-cultural differences in depression may result in a lower prevalence. Moreover, the difference in prevalence across studies may have resulted from different scales and cutoff points. In fact, commonly used scales have fewer common factors for depressive symptoms than expected.^[39]^ The differences in prevalence across studies may vary due to different curricula. Regardless of these differences, our results indicated that many medical students suffer from depression.

Among the sociodemographic factors, female sex and school year (1st and 2nd years) were significantly associated with higher levels of depression. Female sex has been reported as a major risk factor for depression among medical students and physicians.^[40]^ This is consistent with a higher lifetime prevalence of depression among female in the general population.^[41]^ Additionally, previous studies on quality of life have reported that psychological health is poorer in female students than in male students,^[42]^ suggesting that female students are more susceptible to the stressful nature of medical education. Abuhammad and Hamaideh^[43]^ emphasized the role of sex in psychological help-seeking behaviors among international undergraduate nursing students. They found that female students were more willing to seek help for psychological distress than male students, despite experiencing higher perceived stress. While our study similarly found higher levels of depressive symptoms among female students, Korean medical students, regardless of sex, were generally less likely to seek psychiatric support.^[36]^ This contrast may reflect cultural differences in mental health stigma, which tends to be stronger in East Asian countries like Korea. In our study, preclinical years were found to be independent predictors of higher severity of depression. The prevalence of depression was also the highest among 1st-year students (n = 27, 22.5%), followed by 2nd- (n = 17, 18.0%), 3rd- (n = 6, 6%), and 4th-year (n = 5, 5.3%) students. Consistent with our results, a meta-analytic study found that 1st-year students showed the highest rates of depression, which gradually decreased until the 5th year.^[44]^ Prior longitudinal data showed that depressive symptoms of medical students significantly increased from premedical school, suggesting that the higher prevalence in 1st-year students may be attributable to adjustment issues.^[45]^ In Korean medical schools, the 1st year is a transitional period during which students develop the ability to cope with an abrupt increase in their academic workload. Most failures in Korean medical schools occur during the 1st year. Meanwhile, although the difference was insignificant, the prevalence of depression was the highest among 3rd-year medical students in China.^[46]^ Different curricula and related issues may partially explain this discrepancy. Taken together, our results highlight that special attention should be paid to female students and those undergoing preclinical education in order to prevent and manage depression.

According to our findings, medical school authorities should evaluate students’ underlying depression to identify depression early and provide intervention programs. In particular, active efforts to detect and treat depression should be encouraged among female students and those undergoing preclinical education. Moreover, intervention strategies to enhance self-esteem, ego-resiliency, and social support should be considered when developing and implementing intervention programs for depression, as the improvement of these positive psychosocial factors is crucial in alleviating depressive symptoms via direct beneficial and modulating effects. Evidence-based strategies from international studies can be adapted to the Korean educational context. For example, Alhawatmeh et al^[47]^ found that guided imagery and progressive relaxation significantly reduced both physical and emotional symptoms among nursing students undergoing initial clinical training. Such mind–body interventions may be particularly effective for Korean medical students, who often present with somatic expressions of psychological distress. Culturally adapted, relaxation-based programs implemented during the preclinical years could help students develop emotion regulation and coping skills before clinical exposure. Additionally, a previous study demonstrated that attending a mental health course significantly improved students’ attitudes toward seeking professional psychological help.^[48]^

These findings have important implications for mental health policies in Korean medical schools. First, the relatively high prevalence and low utilization of psychiatric services among medical students suggests the need for comprehensive anti-stigma initiatives. Medical schools should implement anti-stigma campaigns, such as student-led discussions and faculty-endorsed statements to normalize mental health concerns. These programs can be incorporated into orientation sessions and reinforced throughout the curriculum. Second, curriculum modifications aimed at reducing unnecessary psychological burden should be considered. For example, academic overload in the 1st and 2nd years may be addressed through more flexible course designs, a pass/fail grading system, and improved feedback structures. Third, institutions should develop targeted psychological support services. Incorporating mental health education into the curriculum may increase students’ willingness to seek assistance.

Despite its meaningful implications, this study had several limitations. First, its cross-sectional design limits causal inference; it remains unclear whether low self-esteem and ego-resiliency contribute to, or result from, depression. Future longitudinal studies are needed to clarify these temporal relationships. Second, the study was conducted at a single medical school, which may limit the generalizability of the findings. Institutional factors, such as curriculum design, faculty culture, and student support services, can vary widely across schools. This context specificity may have biased the observed associations, particularly if the institution had unique protective or risk-promoting characteristics. Third, there is a potential for sampling bias, as participants were selected using a convenience sampling method. Students with severe depression might have avoided participation because of stigma or lack of motivation, which could have led to an underestimation of the true prevalence. Fourth, several potentially important confounding variables, such as academic workload, sleep patterns, coping strategies, family expectations, and prior psychiatric history, were not measured. The exclusion of these factors increases the risk of residual confounding, whereby unaccounted variables influence both psychosocial measures and depressive symptoms, biasing the observed relationships. Fifth, the study employed a cutoff score of 16 on the BDI to classify depression. Although widely used, this threshold may lack diagnostic precision and could have introduced misclassification bias, affecting both prevalence estimates and group comparisons. Finally, the reliance on self-reported questionnaires raises the possibility of reporting bias; students with depressive symptoms may have rated their psychosocial traits more negatively due to cognitive distortions, leading to an overestimation of the association between depression and low self-esteem or social support.

## 5. Conclusions

Self-esteem, ego-resiliency, and social support were inversely related to depression, whereas female sex and preclinical school years were positively associated with depression among medical students. Our study also identified the current status of depression among Korean medical students. Our results may provide valuable information for the faculty and staff members of medical schools responsible for managing depression among medical students. Furthermore, our findings suggest that integrative programs focusing on improving positive psychosocial factors may contribute to depression prevention among medical students.

## Acknowledgments

The authors would like to thank the students who participated in the study.

## Author contributions

**Conceptualization:** Eun Hyun Seo, Hyung-Jun Yoon.

**Data curation:** Eun Hyun Seo, Hyung-Jun Yoon.

**Formal analysis:** Eun Hyun Seo, Hyung-Jun Yoon.

**Funding acquisition:** Hyung-Jun Yoon.

**Project administration:** Hyung-Jun Yoon.

**Supervision:** Seung-Gon Kim, Seon-Cheol Park.

**Writing – original draft:** Eun Hyun Seo, Hyung-Jun Yoon.

**Writing – review & editing:** Eun Hyun Seo, Seung-Gon Kim, Seon-Cheol Park, Hyung-Jun Yoon.
